# Pentraxin 3 is a diagnostic and prognostic marker for ovarian epithelial cancer patients based on comprehensive bioinformatics and experiments

**DOI:** 10.1186/s12935-021-01854-7

**Published:** 2021-04-06

**Authors:** Xiaoying Chang, Dan Li, Chang Liu, Zhe Zhang, Tao Wang

**Affiliations:** 1 Department of Pathology, Shengjing Hospital of China Medical University, 36 Sanhao Street, Heping, Shenyang, 110004 China; 2 Department of Pathology, Shenyang KingMed Center for Clinical Laboratory Co., Ltd, Shenyang, 110164 China

**Keywords:** Ovarian epithelial cancer, WGCNA, Prognosis, Biomarker, PTX3

## Abstract

**Background:**

Ovarian epithelial cancer is one of the leading malignant tumors in gynecology and lacks effective diagnostic and prognostic markers. Our study aims to screen and verify ovarian epithelial cancer biomarkers.

**Methods:**

GSE18520 and GSE26712 were downloaded from the GEO database. The “limma” and “WGCNA” packages were used to explore hub genes. The Kaplan–Meier Plotter database was used for survival analysis of the hub genes. Immunohistochemical analysis was used to identify the expression level of Pentraxin 3 in ovarian epithelial cancer samples.

**Results:**

In this study, we integrated and analyzed two datasets, GSE18520 and GSE26712, and a total of 238 differentially expressed genes (DEGs) were screened out. Enrichment analysis showed that these DEGs were related to collagen-containing extracellular matrix and other pathways. Further application of WGCNA (weighted gene coexpression network analysis) identified 15 gene modules, with the purple module showing the highest correlation with ovarian epithelial cancer. Twenty-five genes were shared between the purple module and DEGs, 13 genes were related to the prognosis of ovarian epithelial cancer patients, and the PTX3 gene had the highest hazardous risk (HR) value. We performed immunohistochemical analyses on the 255 Pentraxin-3 (PTX3)-based clinical samples. PTX3 was found to be overexpressed in ovarian epithelial cancer and related to the degree of differentiation. The Cox proportional hazard model indicates that high PTX3 expression is an independent risk factor for the prognosis of ovarian epithelial cancer patients.

**Conclusions:**

In conclusion, through WGCNA and a series of comprehensive bioinformatics analyses, PTX3 was first identified as a novel diagnostic and prognostic biomarker for ovarian epithelial cancer.

## Introduction

Ovarian cancer is one of the leading malignant tumors in gynecology, ranking first in mortality. Due to the lack of typical symptoms and effective biomarkers in the early stage, approximately 70 % of patients are in an advanced stage when diagnosed clinically [1]. Epithelial ovarian tumors are the main pathological type, accounting for 80–95 % of ovarian cancers [2]. Epithelial ovarian cancer patients are sensitive to chemotherapeutic drugs, and most advanced patients can often achieve clinical remission after standard treatment, but they will eventually succumb to the cancer due to relapse and drug resistance [3, 4]. Therefore, it is of great clinical interest to explore early diagnostic and prognostic biomarkers of ovarian epithelial cancer.

With the development of gene chips and high-throughput sequencing technologies, bioinformatics analysis of gene expression profiles has been widely used to explore potential diagnostic markers or therapeutic targets. Weighted gene coexpression network analysis (WGCNA) is a powerful tool that can be used to describe related patterns between genes and explore central genes related to certain traits [5, 6]. WGCNA divides genes into several coexpression modules by constructing a coexpression network between genes. Finally, the modules are correlated with clinical features to further analyze the modules that are highly relevant to the disease and determine the core genes that are critical for disease progression. WGCNA has been widely used to study cancer [7, 8], chronic diseases [9] and many other diseases. In this study, we used the algorithm to identify core genes related to disease progression.

The pentraxin protein family is a superfamily of evolutionarily conserved proteins and is an important part of the humoral immunity of the innate immune system. It contains multiple members [10, 11]. Among them, pentraxin 3 (Pentraxin 3, PTX3) is a typical acute-phase protein that can be synthesized by a variety of cells at the site of inflammation [12, 13]. Chronic inflammation is a key factor in the development and progression of cancer, suggesting that PTX3 may play an important role in cancer. In recent years, many studies have shown that PTX3 is overexpressed in solid tumors such as liver cancer [14], colon cancer [15], and glioma [16]. Additionally, PTX3 participates in regulating malignant biological behaviors such as proliferation, metastasis, and angiogenesis. However, the role of PTX3 in ovarian epithelial cancer is still unclear.

In this study, we identified modules related to ovarian epithelial cancer by combining difference analysis and the WGCNA coexpression network. After systematic analysis of ovarian cancer-related coexpression modules through a series of bioinformatics methods, PTX3 was confirmed to be a biological marker of ovarian epithelial cancer. PTX3 may become a new diagnostic and prognostic marker of ovarian epithelial cancer in the future.

## Materials and methods

### Data downloading and processing

We selected two ovarian epithelial cancer datasets from the GEO database: GSE18520 and GSE26712. The GSE18520 dataset is based on the GPL570 platform (HG-U133_Plus_2; Affymetrix Human Genome U133 Plus 2.0 Array) and contains 53 high-grade serous papillary carcinoma samples and 10 paracancerous samples. The GSE26712 dataset is based on the GPL96 platform (HG-U133A; Affymetrix human genome U133A array) and contains 10 normal ovarian epithelial samples and 185 primary ovarian epithelial cancer samples. We used batch normalization to correct the data of the two chips, and all the data were normalized.

### Screening for differentially expressed genes (DEGs)

By using the R package “limma”, 20 normal ovarian samples and 238 ovarian epithelial cancer samples were analyzed for differences. | logFC |> 1.5, and p-values < 0.05 were defined as DEGs.

### GO and KEGG analyses

By using the R package “cluster profiler”, a total of 100 upregulated genes and 228 downregulated genes were analyzed by Gene Ontology (GO) enrichment and Kyoto Encyclopedia of Genes and Genomes (KEGG) pathway analyses. A P value < 0.05 was defined as a meaningful enrichment analysis result. GO and KEGG pathway analyses were used to predict potential functions.

### Construction of the weighted coexpression network

The WGCNA software package was used to construct a gene coexpression network. The top 25 % of the genes in the variance map were selected to construct a weighted coexpression network. The network module was subdivided using the dynamic cut tree algorithm. To test the stability of each identified module, the training and test sets were randomly generated using the preservation function module stability in the WGCNA software package. We searched for key modules by evaluating the correlation between modules and clinical features through Pearson-related tests. The clinical features of our samples included normal tissues and ovarian cancer, and we calculated the correlation between modules and features. Modules positively related to ovarian cancer are thought to play a role in the pathogenesis of the disease. On the other hand, genes in modules positively related to normal traits are essential for maintaining normal biological functions. Therefore, we extracted the most relevant gene modules for ovarian cancer for subsequent research.

### Hub gene screening and survival analysis

After combining DEGs and modular genes, we selected overlapping genes as hub genes. The ovarian cancer data in the Kaplan–Meier Plotter database () were used for survival analysis of the hub genes.

### Specimen collection from patients

A total of 255 clinical samples were collected in the Department of Obstetrics and Gynecology, Affiliated Shengjing Hospital to China Medical University. All patients were informed of the experiment and signed an informed consent form. This study was approved by the Clinical Research Ethics Committee of Shengjing Hospital Affiliated to China Medical University. All samples were embedded in paraffin before sectioning. The 215 samples included 168 malignant epithelial ovarian cancer samples, 37 borderline epithelial ovarian tumor samples and 40 normal epithelial ovarian samples. All tumor samples were classified as primary epithelial ovarian tumors. Complete clinical and pathological information of all patients is available. No patient received chemotherapy or hormone therapy prior to surgery.

### Immunohistochemistry

Rabbit anti-human PTX3 antibody was purchased from Abcam. Immunohistochemistry (IHC) kits and DAB staining reagents were purchased from Solarbio. After 10 % formalin fixation, the paraffin-embedded ovarian tissue was sliced into 4 µm sections. The sections were dewaxed with gradient ethanol, blocked with endogenous peroxidase, and then subjected to antigen heat repair treatment by heating in a microwave and naturally cooling to room temperature. The sections were incubated and blocked in goat serum at 37  °C and then incubated in rabbit anti-human PTX3 antibody (1:200 dilution, ab90806, Abcam) at 4 °C overnight. Next, the slices were incubated in horseradish-labeled goat anti-rabbit secondary antibody at 37 °C and stained with 3,3’-diaminobenzidine. Hematoxylin was applied to stain the nucleus blue. The slices were then dehydrated, washed with xylene and fixed. Phosphate-buffered saline was used instead of antibodies as negative controls. Each section was independently evaluated and scored by two pathologists. A semiquantitative scoring system was used in this measurement. The staining intensity was divided into “0” (negative), “1” (weak), “2” (moderate) and “3” (strong). We also calculated the proportion of positively stained tumor cells in each section. Finally, by multiplying the proportional score with the staining intensity score, a final IHC score was calculated. IHC scores ranged from 0 (lowest) to 300 (highest). Positive expression of PTX3 was defined as a detectable immune response with an IHC score > 10.

## Results

### DEG screening

**Fig. 1:**
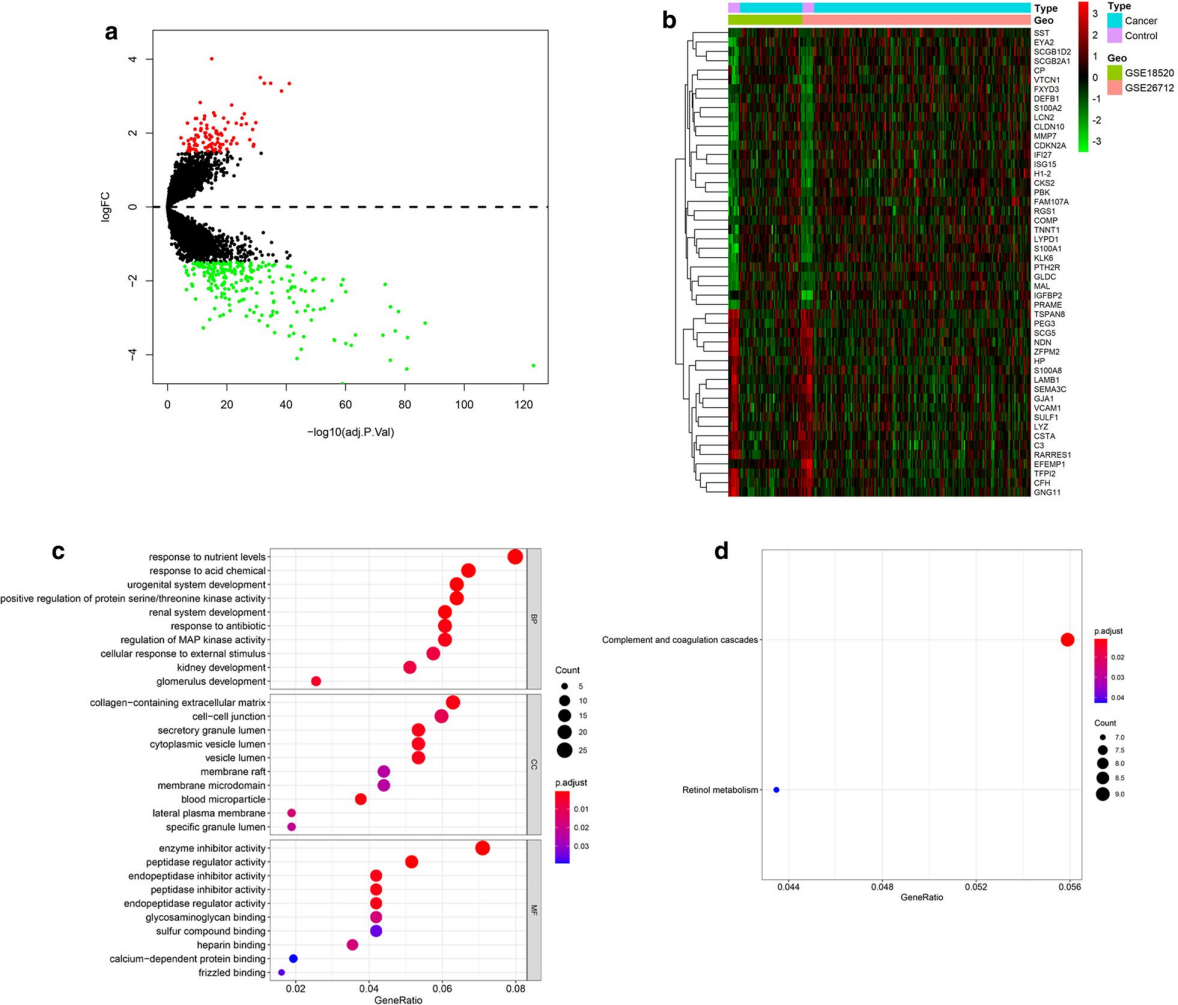
Expression values between DEGs and the enrichment analysis results. **a** Ovarian cancer and normal tissues identified from GSE18520 and GSE26712. Red represents upregulation, and blue shows downregulation. **b** Top 50 differential genes heat map. **c** GO enrichment analyses of DEGs. **d** KEGG enrichment analysis of DEGs

The derivation cohort contained 20 normal ovarian samples and 238 ovarian epithelial cancer samples. Data before and after normalization were visualized via boxplots and examined by principal component analysis (PCA). These results suggested that a batch effect from different datasets was successfully removed (Additional file 1: Figure S1). The expression of the derivation cohort was compared and analyzed using the R package “limma”. A total of 328 DEGs were obtained following p-value < 0.05 and |logFC|≥ 1.5. DEGs are shown in the volcano plot and heat map in Fig. 1a and b.

A total of 328 DEGs were analyzed by Gene Ontology (GO) enrichment using the R package “cluster profiler”. The most enriched GO term based on the DEGs in the biological process (BP) category was “response to nutrient levels”, in the cellular component (CC) category was “collagen-containing extracellular matrix”, and in the molecular function (MF) category was “enzyme inhibitor activity”. KEGG pathway enrichment analysis results are shown in Fig. 1d, which contained “complement and coagulation cascades” and “retinol metabolism”.

### WGCNA

**Fig. 2:**
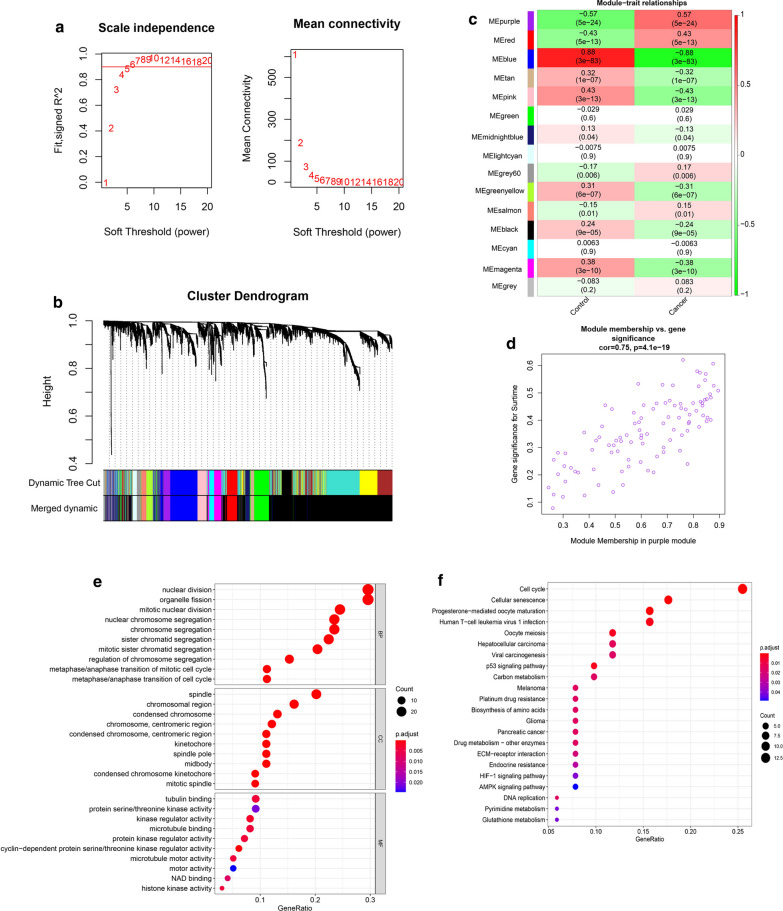
WGCNA. **a** Analyze the scale-free fit index and average connectivity of the 1–20 soft threshold power (β). **b** Genes are grouped into various modules by hierarchical clustering, and different colors represent different modules. **c** Heatmap shows correlations of module eigengenes with features. **d** A scatter plot of the genes in the purple module. **e** GO enrichment analyses of genes in purple module. **f** KEGG enrichment analysis of genes in the purple module

The top 25 % of the genes in the variance plot were screened to construct a coexpression network using the R package “WGCNA”. To build a scale-free network, we picked β = 5 as the soft-thresholding power (Fig. 2a). A total of 15 modules (Fig. 2b) were obtained according to the scale-free network. Among the 15 modules, the purple module was highly correlated with cancer (R^2^ = 0.57, P = 5e−24). The correlation between other modules and cancer was less than 0.5. Therefore, we focused on the purple module as a hub module. A scatter plot of the genes in the purple module is shown in Fig. 2d.

The enrichment analysis results of the purple module are shown in Fig. 2e and f. The most enriched GO term based on the DEGs in BP was “nuclear division”, in CC was “spindle”, and in MF was “tubulin binding”. KEGG pathway enrichment analysis results included “Cell cycle” and “p53 signaling pathway”, which are both well-known pathways.

### Survival analysis of the hub genes

**Fig. 3:**
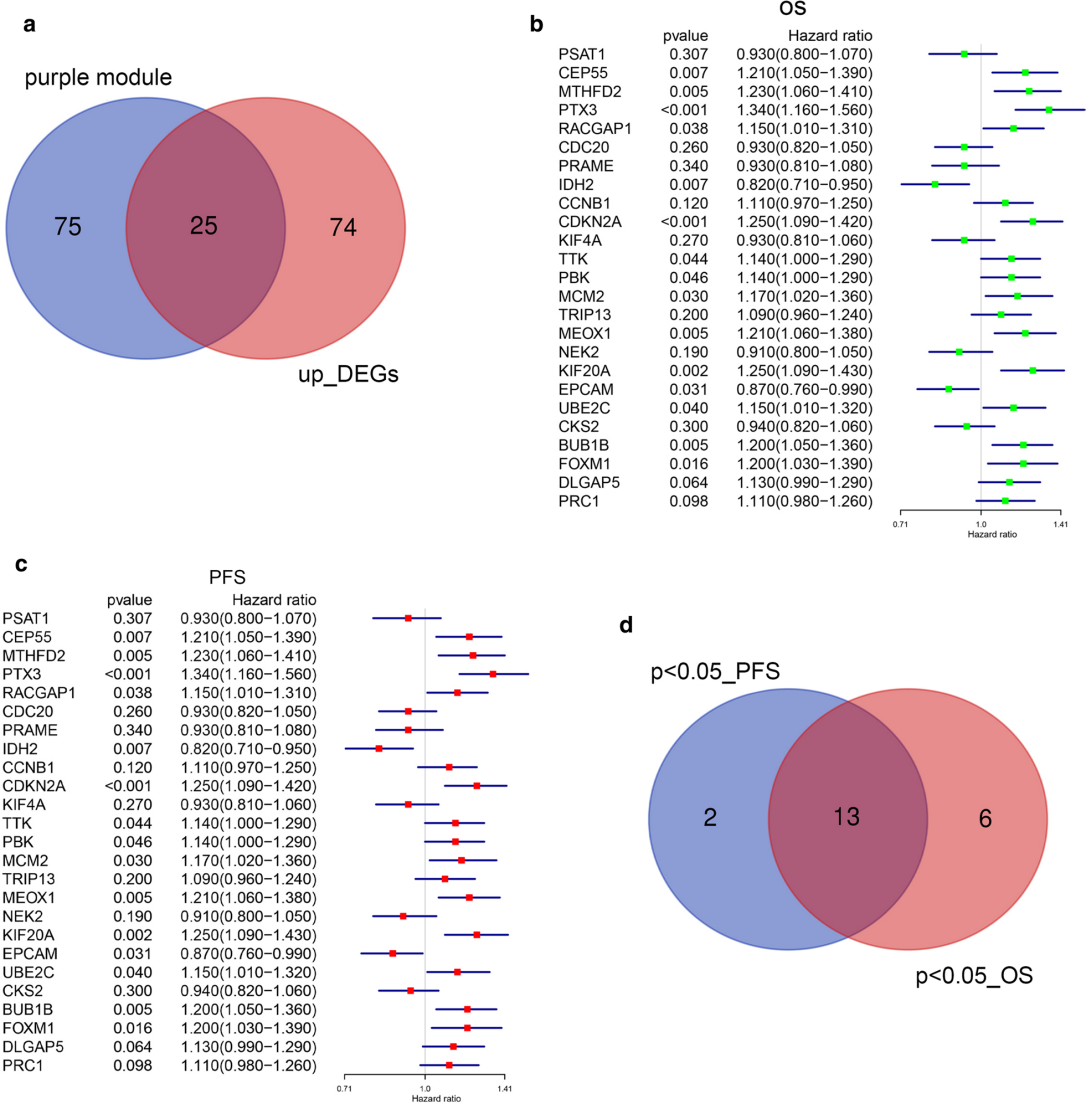
Survival analysis of hub genes. **a** up_DEGs and module gene Venn diagram. **b** Prognostic values of 25 genes in ovarian tumors (OS in Kaplan–Meier plotter). **c** Prognostic values of 25 genes in ovarian tumors (PFS in Kaplan–Meier plotter). **d** Genes were significantly different in both OS and PFS

Twenty-five genes, which were identified as both upregulated DEGs and purple module genes, were selected and designated hub genes (Fig. 3a). The survival value of hub genes was explored from the KM-PLOTTER database. An OS (overall survival) forest map of these genes is shown in Fig. 3b, and the progression-free survival (PFS) forest map of these genes is also shown in Fig. 3c. Thirteen genes (BUB1B, KIF20A, MCM2, CEP55, MTHFD2, FOXM1, PBK, CDKN2A, PTX3, RACGAP1, MEOX1, UBE2C, IDH2) were associated with the OS and PFS of ovarian epithelial cancer patients (Fig. 3d).

### Relationship between PTX-3 expression and the clinical pathological features of ovarian epithelial cancer

**Fig. 4:**
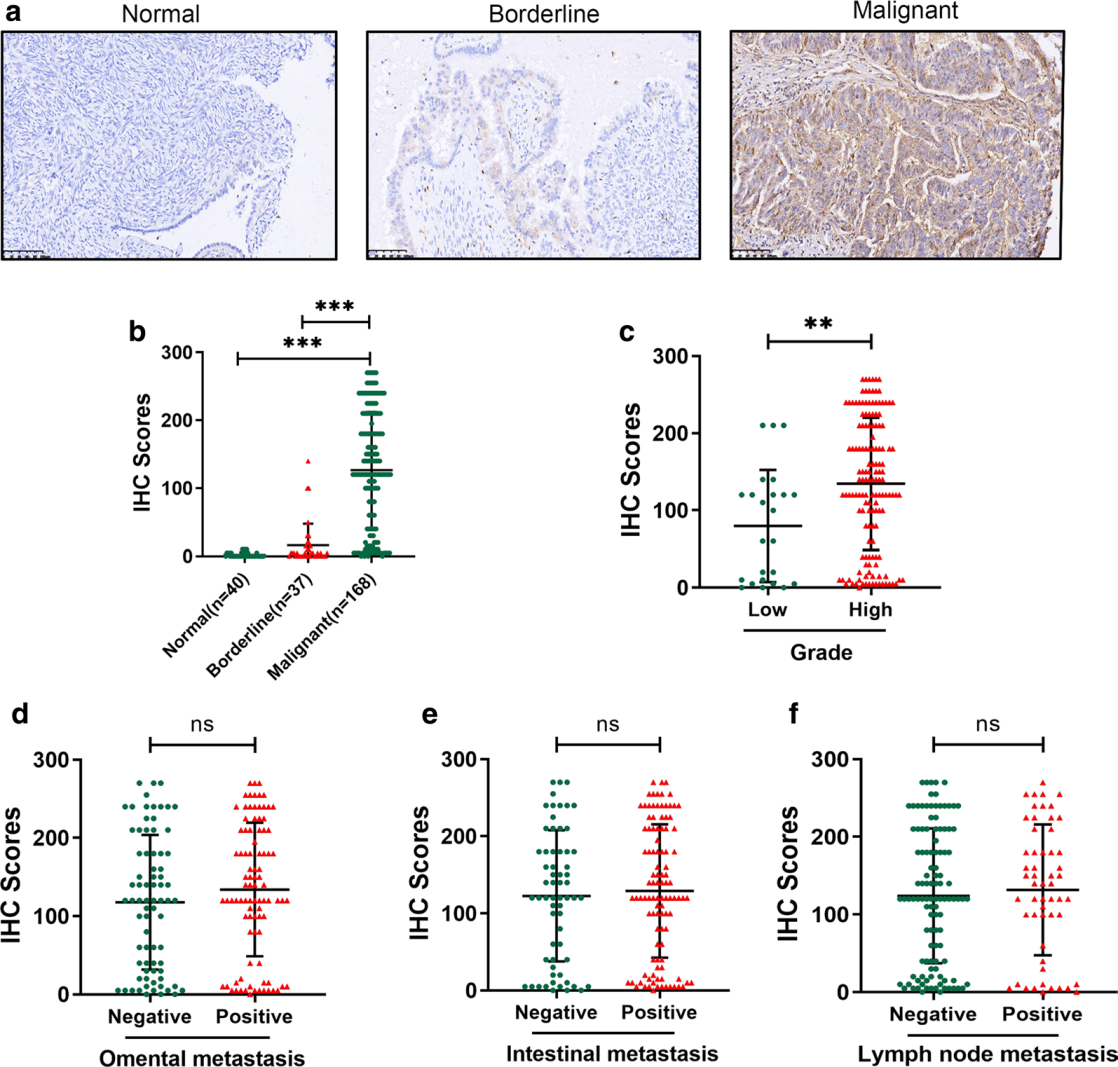
PTX3 protein expression was overexpressed in ovarian tumor tissues. **a** Representative images of PTX3 staining in human normal ovary tissue, borderline tissue and malignant ovarian tumor; **b** The IHC scores of PTX3 were significantly increased in malignant ovarian tissues compared to ovary tissues and borderline tissues; **c** The IHC scores of PTX3 were increased in high-grade compared with low-grade patients; **d**–**f** The IHC scores of PTX3 show no significant difference in differential status of omental metastasis, intestinal metastasis and lymph node metastasis

Thirteen genes from normal ovary epithelial tissues and ovarian epithelial cancer tissues were analyzed via PCR. Additional file 2: Fig. S2 shows that the change in PTX3 was most obvious. PTX3 has a higher hazardous risk (HR) value, and no previous studies have shown its relationship with ovarian epithelial cancer. We chose PTX3 for the next experiment. Immunohistochemical assays of PTX-3 expression in normal ovary epithelial tissue, borderline tissue and malignant tumors are shown in Fig. 4a. The IHC scores of PTX-3 in malignant tumors were significantly higher than those in normal ovarian epithelial and borderline tissues (Fig. 4b). The IHC scores of PTX-3 in high-grade malignant tumors were significantly higher than those in low-grade malignant tumors (Fig. 4c). However, no significant differences in IHC scores were observed among omental metastasis, intestinal metastasis, and lymph node metastasis (Fig. 4d–f).

### Diagnostic and prognostic value of PTX-3 in ovarian epithelial cancer

**Fig. 5:**
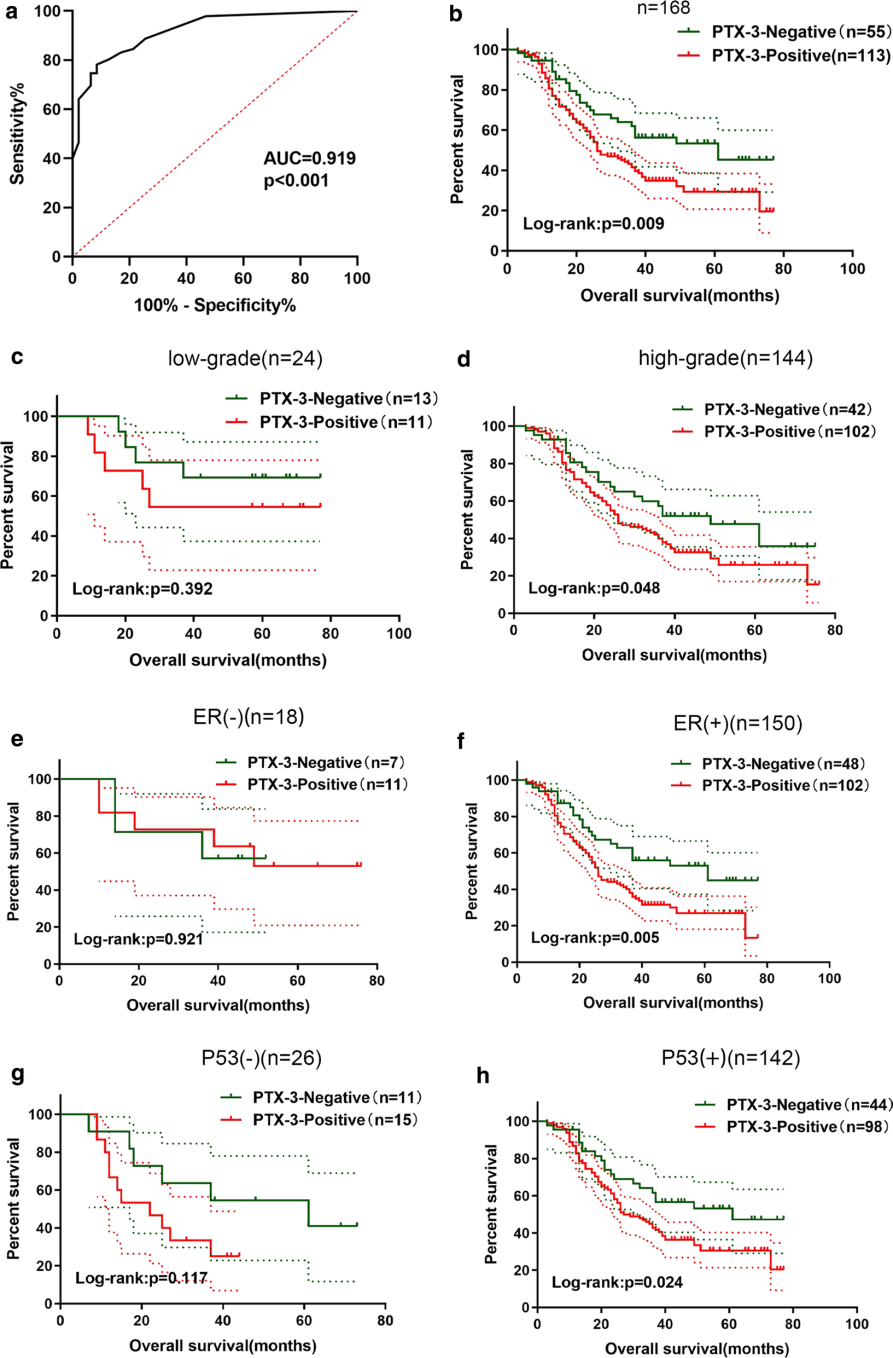
Diagnostic and survival value of PTX3 in ovarian epithelial cancer. **a** ROC analysis for PTX3 diagnostic value in ovarian epithelial cancer; **b** Kaplan–Meier analysis for overall survival in ovarian epithelial cancer patients; **c** Kaplan–Meier analysis for overall survival in low-grade ovarian epithelial cancer patients; **d** Kaplan–Meier analysis for overall survival in high-grade ovarian epithelial cancer patients; **e** Kaplan–Meier analysis for overall survival in ER(−) ovarian epithelial cancer patients; **f** Kaplan–Meier analysis for overall survival in ER(+) ovarian epithelial cancer patients; **g** Kaplan–Meier analysis for overall survival in P53(−) ovarian epithelial cancer patients; **h** Kaplan–Meier analysis for overall survival in P53(+) ovarian epithelial cancer patients

Receiver operating characteristic (ROC) curves were carried out to further evaluate the diagnostic value of PTX-3 in ovarian epithelial cancer. As Fig. 5a shows, PTX-3 expression could be distinguished between ovarian epithelial cancer tissue, normal ovary epithelial tissue and borderline tissue (AUC = 0.919, p < 0.001). The results of Kaplan–Meier analysis showed that the OS of PTX3-positive patients was significantly lower than that of PTX3-negative patients (p = 0.009, Fig. 5b). A further subgroup analysis showed that higher PTX3 expression was associated with the poor prognosis of patients with high-grade ovarian epithelial cancer but was not related to the prognosis of patients with low-grade ovarian epithelial cancer (Fig. 5c, d). Further results showed that the expression of PTX3 was related to the prognosis of ER(+) and P53(+) ovarian epithelial cancer patients (Fig. 5e–h).

**Fig. 6:**
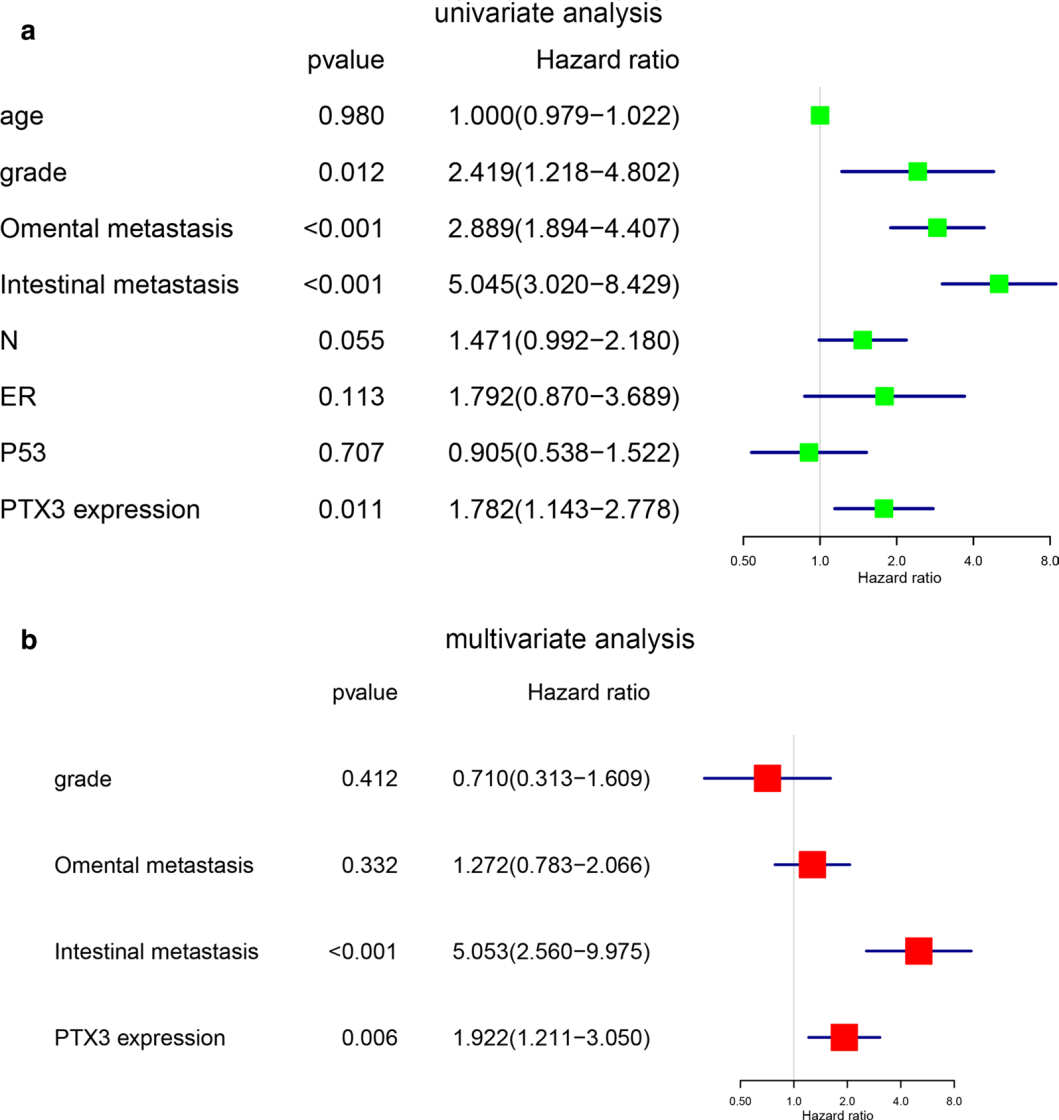
Relationship of PTX3 expression with ovarian epithelial cancer prognosis. **a** Forest map based on univariate Cox regression analysis of the OS of ovarian epithelial cancer patients. **b** Forest map based on multivariate Cox regression analysis of the OS of ovarian epithelial cancer patients

The relationship between age, grade, omentum metastasis, intestinal metastasis, lymph node metastasis, ER, P53, PTX3 expression levels and survival time of ovarian epithelial cancer patients was analyzed by a Cox regression model. The results of univariate analysis showed that grade (HR = 2.419, 95 % HR = 1.128–4.802, p = 0.012), omental metastasis (HR = 2.889, 95 % HR = 1.894–4.407, p < 0.001), intestinal metastasis (HR = 5.045, 95 % HR = 3.020–8.429, p < 0.001), and PTX3 expression (HR = 1.782, 95 % HR = 1.143–2.778, p = 0.011) were significantly correlated with overall survival (Fig. 6a). Further multivariate analysis revealed that intestinal metastasis (HR = 5.053, 95 % HR = 2.560–9.975, p < 0.001) and PTX3 expression levels (HR = 1.922, 95 % HR = 1.211–3.050, p = 0.006) were independent risk factors (Fig. 6b).

### Validation of the diagnostic and prognostic performance of the PTX-3 gene in an external GEO dataset

**Fig. 7:**
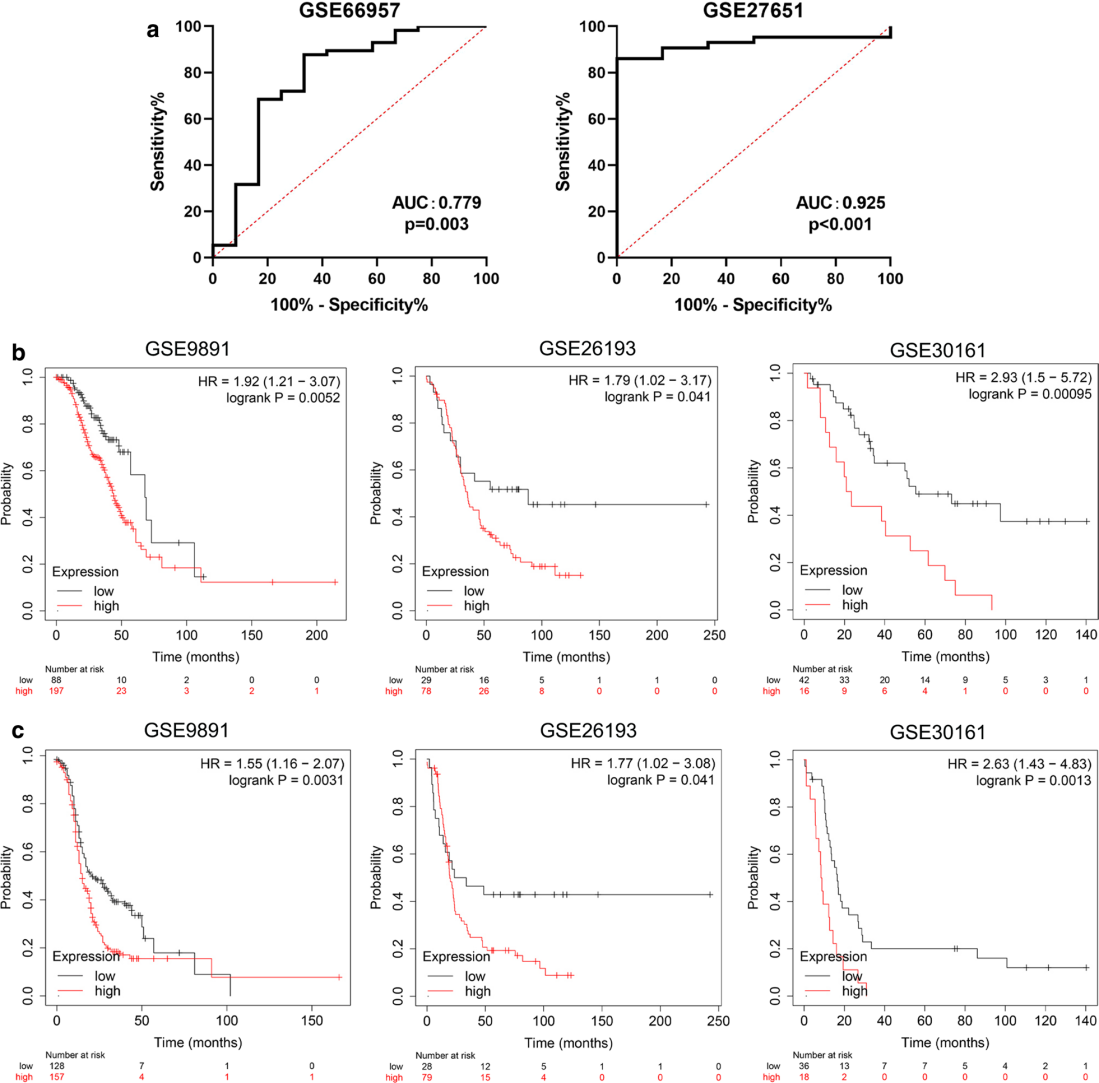
Validation of the diagnostic and prognostic performance of the PTX-3 gene in an external GEO dataset. **a** ROC analysis for PTX3 diagnostic value in ovarian epithelial cancer based on GSE66957 and GSE27651; **b** prognostic values (OS) of the PTX-3 gene in ovarian tumors based on GSE989, GSE26193 and GSE30161; **c** prognostic values (PFS) of the PTX-3 gene in ovarian tumors based on GSE989, GSE26193 and GSE30161

To further verify the diagnostic and prognostic performance of the PTX-3 gene in an external GEO dataset, first, we selected two GEO datasets (including normal ovarian epithelial samples and ovarian epithelial cancer epithelial samples) to verify the diagnostic efficacy of PTX3 (Fig. 7a). In GSE66957 (12 normal ovarian epithelial samples, 57 ovarian epithelial tumor samples), the AUC value of PTX3 to predict ovarian epithelial cancer was 0.779 (p = 0.003). In GSE27651 (6 normal ovary epithelial samples, 43 ovarian tumor samples), the AUC value of PTX3 was 0.925 (p < 0.001). To verify the prognostic performance of the PTX-3 gene, we chose three GEO datasets (both have OS and PFS follow-up data). In GSE9891, the PTX3 level was associated with the OS (HR = 1.92, p = 0.0052) and PFS (HR = 1.55, p = 0.0031) of ovarian epithelial cancer patients. In GSE26193, the PTX3 level was associated with the OS (HR = 1.79, p = 0.041) and PFS (HR = 1.72, p = 0.041) of ovarian epithelial cancer patients. Finally, in GSE30161, the PTX3 level was also associated with the OS (HR = 2.93, p = 0.00095) and PFS (HR = 2.63, p = 0.0013) of ovarian epithelial cancer patients. These results further confirmed the diagnostic and prognostic value of the PTX3 gene.

## Discussion

In this study, we used bioinformatics methods to integrate two datasets: GSE18520 and GSE26712. Through differential analysis, 328 DEGs were identified; 100 genes were upregulated, and 228 genes were downregulated. Further combined with GO analysis and KEGG pathway analysis, the “collagen-containing extracellular matrix”, “enzyme inhibitor activity”, “complement and coagulation cascades” and “retinol metabolism” pathways were significantly enriched. These results suggest that these differentially expressed genes may affect the biological behavior of ovarian epithelial cancer cells through those pathways, thereby regulating the occurrence and development of ovarian epithelial cancer.

To identify genes that play a key role in the development of ovarian epithelial cancer, we further applied the WGCNA algorithm to analyze the datasets. WGCNA builds a gene coexpression network, finds a gene module for coexpression, and explores the association between the gene network and phenotype. WGCNA is widely used to explore the pathogenesis of tumors and other diseases. In this study, we divided all genes into 15 coexpression modules through WGCNA. After correlating modules with clinical features, we concluded that the purple module had the highest correlation with ovarian epithelial cancer and was suitable for mining core genes. The enrichment of GO and KEGG indicated that the genes in the purple module were mainly concentrated in pathways such as “nuclear division”, “spindle”, “tubulin binding”, “cell cycle”, and “p53 signaling pathway”. This suggested that during the progression of ovarian epithelial cancer, the tumor cell cycle changed abnormally. In addition, we intersected the genes in the purple module with the upregulated DEGs to obtain 25 core genes. Furthermore, these core genes were combined with the KM-PLOTTER database, and a total of 13 genes, including BUB1B, KIF20A, MCM2, CEP55, MTHFD2, FOXM1, PBK, CDKN2A, PTX3, RACGAP1, MEOX1, UBE2C, and IDH2, were identified. These results suggested that these genes might participate in the regulation of the malignant biological behavior of ovarian epithelial cancer by participating in cell cycle-related pathways, thereby affecting the prognosis of patients.

For example, BUB1B, a kinase involved in spindle checkpoint function, has been shown to be a prognostic marker for ovarian epithelial cancer [17, 18]. KIF20A participates in the processes driving cell division (mitosis) via mitotic spindles [19]. Several studies have recently shown a prominent increase in the level of KIF20A expression in a variety of malignancies, including breast [20], lung [21], liver [22], and gastric cancers [23]. In ovarian clear-cell carcinoma cells, KIF20A may significantly promote the proliferation of ovarian clear-cell carcinoma cells [24]. MCM2, a highly conserved minichromosome maintenance protein (MCM), is involved in the initiation of eukaryotic genome replication and is closely related to the prognosis of ovarian cancer patients and the sensitivity to chemotherapy [25–27]. High expression of the centrosomal protein CEP55 has been correlated with clinicopathological parameters across multiple human cancers, such as thyroid cancer [28], liver cancer [29], lung cancer [30] and epithelial ovarian carcinoma [31]. MTHFD2 is a metabolic enzyme and plays an important regulatory role in tumorigenesis, including acute myeloid leukemia (AML) [32], breast cancer [33], and renal cell carcinoma (RCC) [34]. FOXM1 is a transcription factor that regulates the expression of cell cycle genes essential for DNA replication and mitosis and plays a role in the control of cancer cell proliferation [35, 36]. PBK is a protein kinase related to MAPKK family, and overexpression of PBK has been implicated in tumorigenesis [37, 38]. CDKN2A could induce cell cycle arrest in G1 and G2 phases, and CDKN2A loss has been shown to be a significant event in several cancer types [39, 40]. RACGAP1 encodes a GTPase-activating protein (GAP), binds activated forms of Rho GTPases and stimulates GTP hydrolysis, which results in negative regulation of Rho-mediated signals, promoting malignant progression in gastric cancer [41], breast cancer [42] and epithelial ovarian cancer [43]. MEOX1 plays a role in the molecular signaling network regulating somite development and promotes tumor progression in lung cancer [44] and prostate cancer [45]. UBE2C encodes an E2 ubiquitin-conjugating enzyme, is required for the destruction of mitotic cyclins and for cell cycle progression, and is involved in cancer progression (cervical cancer [46] and ovarian cancer [47, 48]). IDH2 is the NADP-dependent isocitrate dehydrogenase found in mitochondria and plays a role in intermediary metabolism and energy production. IDH2 mutations have been observed in several cancer types, including sarcomas, hematologic malignancies, colon cancer and brain cancer [49–51]. Based on the above data from the literature, these genes may function as potential biomarkers for ovarian cancer management.

Compared with the other 12 genes, PTX3 had the largest upregulation in ovarian epithelial cancer tissues. Further protein level verification of PTX3 shows the clinical value in the diagnosis and prognosis of ovarian epithelial cancer. PTX3 was originally discovered in 1992 and is a member of the orthopentamerin family. It is a typical acute-phase protein that is expressed by hematopoietic cells and stromal cells in response to primary proinflammatory stimuli, and it is essential for innate immune humoral immunity, with involvement in the identification of pathogens, inflammation regulation and tissue remodeling [52–54]. Studies in tumors showed that PTX3 promoted cell migration and invasion, and its expression level was related to the progression of different tumor types in humans. For example, high expression of PTX3 was observed in pancreatic cancer, and there was a strong correlation between the prognosis of pancreatic cancer patients [55]. At the same time, the production of PTX3 promotes the upregulation of epidermal growth factor (EGF) in head and neck squamous cell carcinoma cells, tumor cell migration, invasion and EGF-mediated metastasis [56]. Another study showed that PTX3 expression was increased in gastric cancer tissues and promoted tumor cell migration and macrophage recruitment, leading to gastric cancer-related inflammation [57]. In female reproductive system tumors, PTX3 was found to be overexpressed in cervical cancer, which was related to tumor grade and differentiation. Silencing PTX3 demonstrated a significant inhibition of the malignant biological behavior of cervical cancer cells [58, 59]. However, its role in the ovarian epithelium is still unclear and requires further investigation. In this study, the protein expression levels of PTX3 in normal ovarian epithelial tissues, borderline ovarian tumor samples and malignant ovarian cancer epithelial samples were detected by IHC. The results showed that PTX3 was highly expressed in ovarian epithelial cancer tissues. Further analysis showed that the expression level of PTX3 was significantly correlated with the degree of tumor differentiation. In addition, we tried to analyze the diagnosis and prognostic value of PTX3 in patients with ovarian epithelial cancer. The results showed that we could significantly distinguish malignant tumors from borderline tumors and normal ovarian epithelial tissues based on the expression level of PTX3 (AUC = 0.919, p < 0.001). Furthermore, the results of the analysis by the Kaplan–Meier (KM) method and single-factor and multifactor Cox analyses showed that PTX3 was an independent adverse prognostic factor in patients with ovarian epithelial cancer. Based on the above research results, we first confirmed the diagnostic and prognostic value of PTX3 in ovarian epithelial cancer.

However, the study still has some limitations. First, due to the lack of in vitro or in vivo experiments, the in-depth mechanism of PTX3 in ovarian epithelial cancer needs to be further elucidated. Second, this study is a retrospective study, so prospective studies should be conducted to verify the results.

In conclusion, through WGCNA and a series of comprehensive bioinformatics analyses, PTX3 was first confirmed as a biomarker for ovarian epithelial cancer. PTX3 may become a new diagnostic and prognostic marker of ovarian epithelial cancer.

## Supplementary Information

**Additional file 1: Fig. S1.** Data preprocessing of the derivation cohort. Box plot and principal component analysis showing the overall profiles of GSE18520 and GSE26712 (A, B).

**Additional file 2: Fig. S2.** The expression level of 13 genes in normal ovary epithelial tissues and ovarian epithelial cancer via PCR analysis

## Data Availability

Not applicable.
