# Supplementary figures and images for: Pentraxin 3 is a diagnostic and prognostic marker for ovarian epithelial cancer patients based on comprehensive bioinformatics and experiments
Source: Cancer Cell Int. 2021 Apr 6;21:193. doi: 10.1186/s12935-021-01854-7 (PMC8097951; doi:10.1186/s12935-021-01854-7)

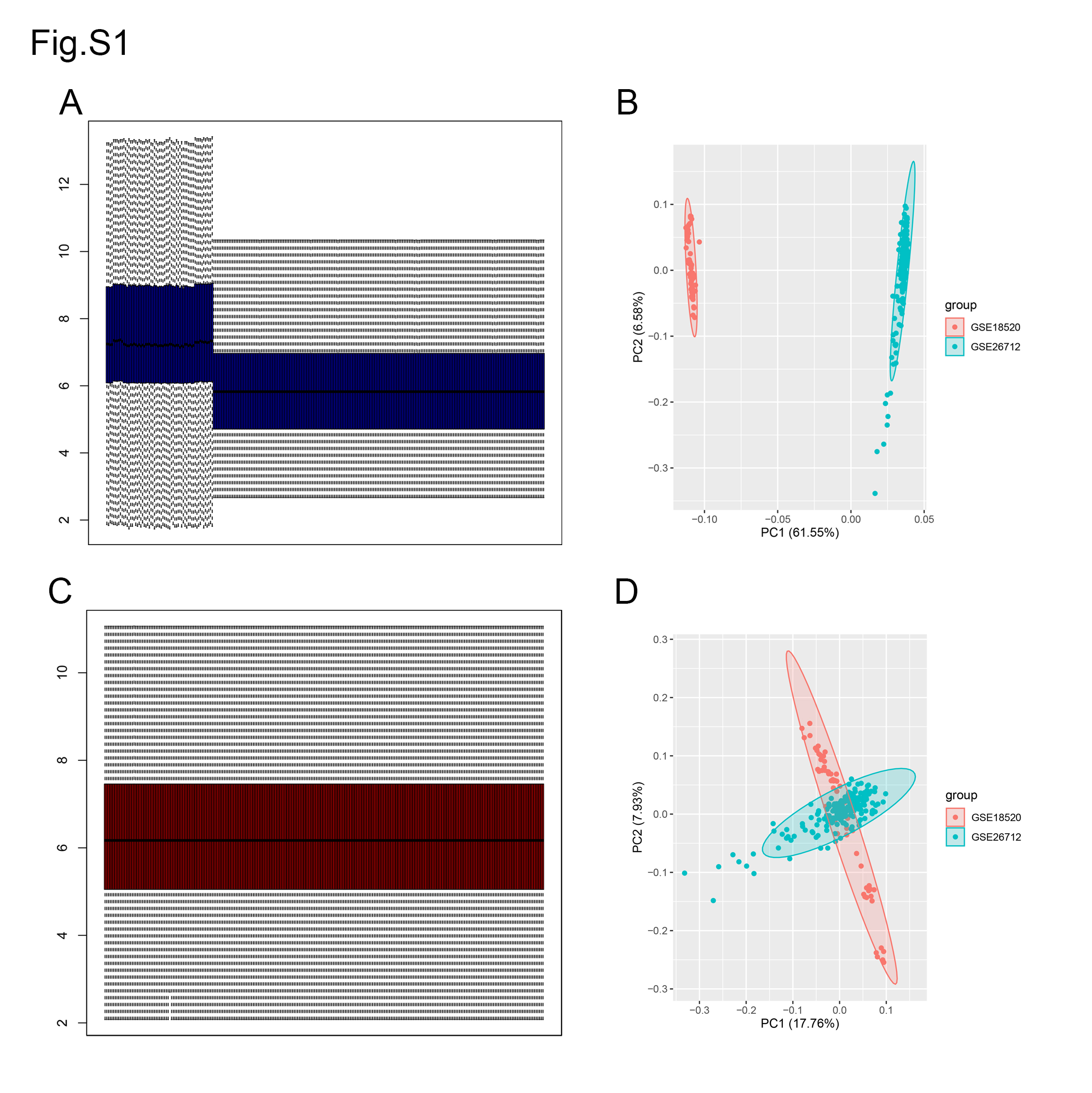

Supplement: Supplementary file 1 — Additional file 1: Fig. S1. Data preprocessing of the derivation cohort. Box plot and principal component analysis showing the overall profiles of GSE18520 and GSE26712 (A, B). [file 12935_2021_1854_MOESM1_ESM.jpg]

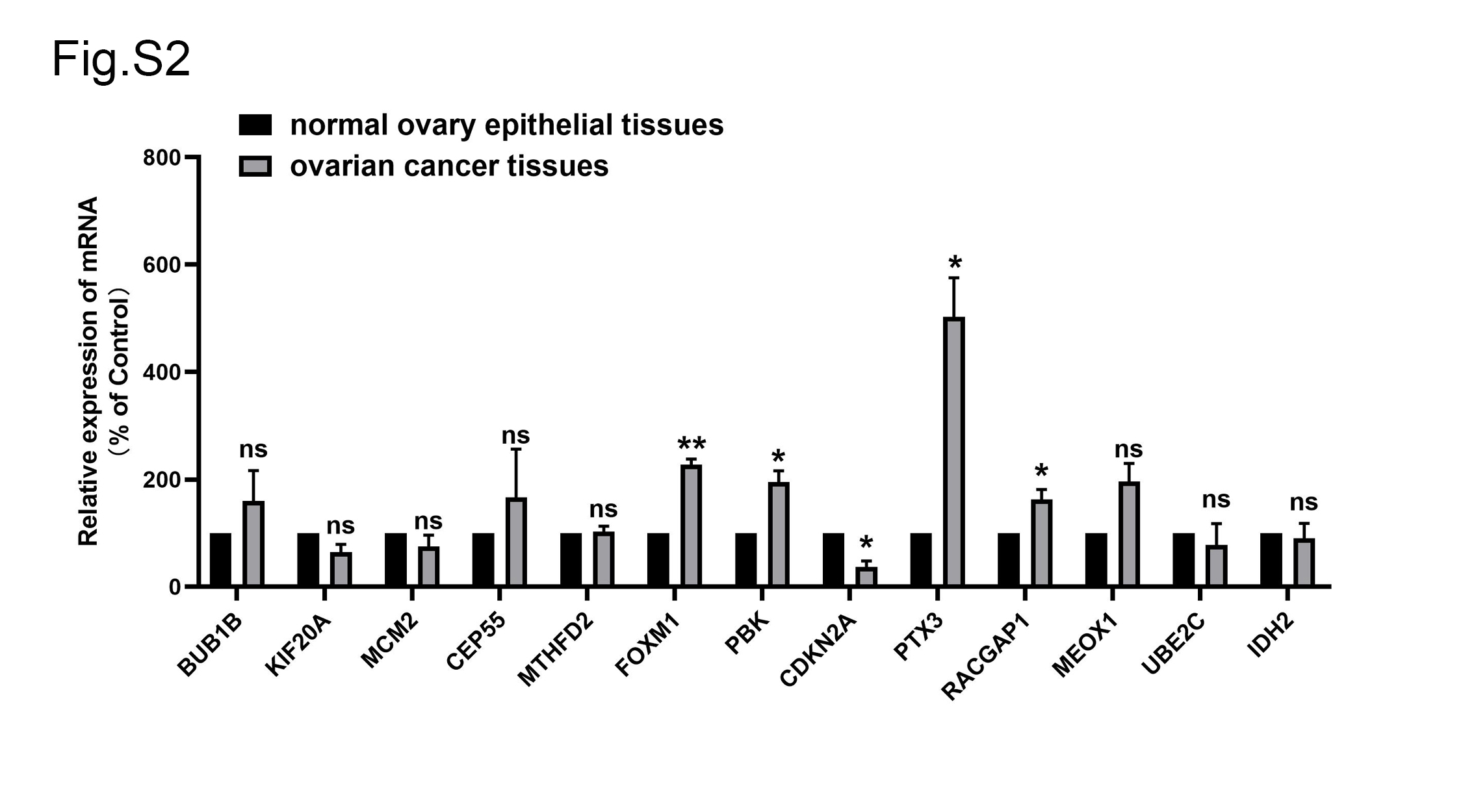

Supplement: Supplementary file 2 — Additional file 2: Fig. S2. The expression level of 13 genes in normal ovary epithelial tissues and ovarian epithelial cancer via PCR analysis [file 12935_2021_1854_MOESM2_ESM.jpg]
